# A single‐center retrospective analysis of prognoses in patients with melanoma brain metastases and effectiveness of treatment in Japan

**DOI:** 10.1002/cam4.6767

**Published:** 2023-12-11

**Authors:** Shogo Wada, Dai Ogata, Tairo Kashihara, Kae Okuma, Hirofumi Eto, Eiji Nakano, Akira Takahashi, Kenjiro Namikawa, Hiroshi Igaki, Naoya Yamazaki

**Affiliations:** ^1^ Department of Dermatologic Oncology National Cancer Center Hospital Tokyo Japan; ^2^ Department of Radiation Oncology National Cancer Center Hospital Tokyo Japan; ^3^ Department of Dermatology, Faculty of Medicine University of Miyazaki Hospital Miyazaki Japan; ^4^ Department of Dermatologic Oncology National Cancer Center Hospital East Chiba Japan

**Keywords:** brain metastasis, immunotherapy, melanoma, prognostic factors

## Abstract

**Background:**

Melanoma brain metastasis (MBM) has a poor prognosis, although recent treatments, including immune checkpoint inhibitors and targeted therapy, have improved the prognosis. However, these systemic therapies have been reported to be less efficient for Asian patients. We investigated the survival of Asian patients with MBM and the effectiveness of systemic therapies.

**Methods:**

We retrospectively reviewed the survival rates of patients diagnosed with MBM between January 2011 and December 2021 at the National Cancer Center Hospital in Tokyo, Japan. In addition, we identified factors associated with survival using Cox regression analysis.

**Results:**

A total of 135 patients were included. The median overall survival (OS) after an MBM diagnosis was 7.8 months (95% confidence interval [CI] 6.1–9.6). The 6‐month and 1‐year survival rates were 60.7% and 34.8%, respectively. We identified the prognostic factors of MBM, including non‐acral primary location, low serum LDH levels, systemic therapy of single‐agent immune checkpoint inhibitors (ICIs) or targeted therapies (TTs), and radiotherapy of stereotactic irradiation (STI). We found no significant difference in effectiveness between single‐agent ICIs, the combination of Nivolumab and Ipilimumab (COMBI‐ICI), and TTs (COMBI‐ICI vs. single‐agent ICI, hazard ratio 0.71, 95% confidence interval 0.27–1.88, *p* = 0.49; COMBI‐ICI vs. TT: hazard ratio 0.46, 95% confidence interval 0.14–1.55, *p* = 0.21).

**Conclusions:**

Systemic therapy and radiotherapy have improved the survival of MBM patients, but the survival of Asian patients remains poor. Our findings suggest that COMBI‐ICIs are not significantly more effective than single‐agent ICI or TT in treating MBM.

## Graphical abstract

Kaplan–Meier survival curves categorized by regimens of initial systemic therapy. There was no significant difference between the three types of systemic therapy.

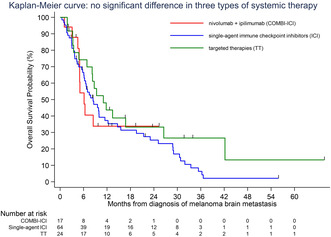

## 1 INTRODUCTION

Melanoma is the third most common source of brain metastases following lung and breast cancer.^1^ Melanoma brain metastases (MBMs) are important targets for treatment because they are associated with significant morbidity. Previously, patients with MBMs had poor prognosis, with an estimated median overall survival (OS) of approximately 4 months.^2^ However, several systemic therapies have evolved and successfully improved outcomes. Immune checkpoint inhibitors (ICIs), such as nivolumab, ipilimumab, and pembrolizumab, have been approved, followed by therapies targeting *BRAF* ^V600^ mutation. Targeted therapies (TTs) include BRAF inhibitors (dabrafenib, vemurafenib, and encorafenib) and MEK inhibitors (trametinib, cobimetinib, and binimetinib). In combination with radiotherapy, these new drugs have successfully improved the outcomes of MBM patients.^3^ Particularly, a combination therapy of nivolumab and ipilimumab (COMBI‐ICI) has been reported to be more effective than single‐agent ICIs and TTs.^4^, ^5^, ^6^, ^7^

Although many prospective and retrospective studies have shown the effectiveness of ICIs and TTs in patients with MBM, the cohorts in these studies were primarily Caucasian, and data regarding the outcomes of Asian patients with MBM are scarce. Furthermore, some studies have shown that ICIs are not as effective in Asians as in Caucasians.^8^, ^9^ However, the effectiveness of ICIs and TTs for Asian patients with MBM has rarely been reported. Therefore, it is important to investigate the effectiveness of ICIs and TTs for Asian patients with MBM to treat them appropriately. This study aimed to elucidate the outcome of MBM in Asian patients and investigate the factors associated with the outcome and the best treatment choice. We emphasize the importance of recognizing treatment effect heterogeneity across different racial groups when treating patients with MBM.

## 2 METHODS

### 2.1 Study design

We retrospectively identified patients diagnosed with MBM at the National Cancer Center Hospital in Tokyo, Japan. Medical records of all patients were reviewed to extract data on demographics, tumors, and treatment characteristics. We included all Asian patients who had been diagnosed with MBM between January 2011 and December 2021. We excluded patients belonging to other ethnicities. In addition, we assessed outcomes, including overall survival (OS) and 6‐month and 1‐year survival rates, and further investigated the association between the characteristics and these outcomes in patients with MBM.

### 2.2 Patient characteristics

Demographic characteristics included age at diagnosis, sex, ethnicity, and pre‐treatment Eastern Cooperative Oncology Group Performance Status (PS). The tumor characteristics included the presence or absence of neurological symptoms, number and size of the MBM, location of the primary tumor, number of extracranial metastatic organs, serum lactate dehydrogenase (LDH) levels, PD‐L1 expression status, and *BRAF* ^V600^ mutational status. The upper limit of the normal serum LDH level was 240 U/L. The number and size of the MBM and number of extracranial metastatic organs were evaluated using computed tomography (CT) scans. The MBM size referred to the largest brain metastasis in each patient. Treatment characteristics included the type of systemic therapy administered first after the diagnosis of MBM and the type of radiotherapy. Systemic therapy options included dacarbazine, combination of carboplatin and paclitaxel, nivolumab, ipilimumab, COMBI‐ICI, combination of dacarbazine and ipilimumab, pembrolizumab, vemurafenib, dabrafenib, trametinib, combination of dabrafenib and trametinib, and combination of encorafenib and binimetinib. All regimens were categorized as single‐agent ICI (nivolumab, ipilimumab, and pembrolizumab), COMBI‐ICIs, TT (vemurafenib, dabrafenib, trametinib, combination of dabrafenib and trametinib, and combination of encorafenib and binimetinib), and chemotherapy (dacarbazine and combination of cisplatin and paclitaxel). Patients treated with combination of dacarbazine and ipilimumab were excluded because the regimen cannot be categorized into them, and it is not officially used now. Radiotherapy included stereotactic irradiation (STI) and whole‐brain radiotherapy (WBRT). STI was delivered at a dose of 9–42 Gy in 1–15 fractions, and WBRT was delivered at a dose of 30–42.5 Gy in 1–13 fractions.

### 2.3 Statistical analysis

We used the Kaplan–Meier method to evaluate OS and the 6‐month and 1‐year survival rates. The OS was calculated from the date of MBM diagnosis to death attributable to melanoma or the last follow‐up before December 31, 2021. Data from patients who did not die were censored on December 31, 2021. The 6‐month survival rate was defined as the rate of survival 6 months after the date of MBM diagnosis. We used the log‐rank test to compare the Kaplan–Meier survival curves. Descriptive statistics are presented as frequencies for categorical variables and medians and ranges for continuous variables. We analyzed the demographic, tumor, and treatment characteristics as prognostic factors for OS using the chi‐square test, Mann–Whitney *U* test, and Cox regression analyses. In addition, we examined the effectiveness of single‐agent ICI, COMBI‐ICIs, and TT in prolonging the survival of patients with MBM using the Kaplan–Meier method and Cox regression analyses. All the tests were two‐sided. Statistical significance was set at *p* < 0.05. Statistical analyses were performed using Stata version 17 (StataCorp LLC). The study protocol was approved by the Institutional Review Board of the National Cancer Center, Japan (approval No. 2013‐081).

## 3 RESULTS

### 3.1 Patient characteristics

Between January 2011 and December 2021, 140 patients were diagnosed with MBM at the National Cancer Center Hospital. Among them, four (2.9%) non‐Asian patients and one (0.7%) patient treated with combination of dacarbazine and ipilimumab were excluded. The demographic, tumor, and treatment characteristics of the remaining 135 patients with MBM are summarized in Table 1. The median follow‐up period was 6.7 months (range, 0.5–67.5 months). The median age at initial MBM diagnosis was 63 (range, 19–87) years (male, 76 [56.3%], female, [43.7%]). The median number of MBM was two (range: 2–113) and the median size of the MBM was 13 mm (range: 1–74). Twenty‐nine (21.5%) and 22 (16.3%) patients had acral and mucosal melanomas, respectively, as the primary locations. The median number of extracranial metastatic organs was 3 (0–8). The median serum LDH level was 246 U/L (131–2840). PD‐L1 expression status was examined in 21 patients (15.6%); the number of patients with PD‐L1 expression >1% and <1% was 12 (8.9%) and 9 (6.7%), respectively. *BRAF* ^V600^ mutational status was examined in 104 patients (77.0%); the number of patients with BRAF wild‐type and BRAF mutant melanoma was 50 (37.0%) and 54 (40.0%), respectively. The BRAF mutations included V600E (*n* = 29, 20.7%), V600K (*n* = 2, 1.5%), and V600R (*n* = 1, 0.7%). All patients with BRAF‐mutant melanoma underwent TTs as first‐line therapy. Systemic therapy was initiated in 114 (84.4%) patients. All the patients received radiotherapy. STI and WBRT were performed in 110 (81.5%) and 25 (18.5%) patients, respectively. There was no significant difference in the number and size of MBM between patients who underwent STI and WBRT (the number, *p* = 0.31; the size, *p* = 0.41).

**TABLE 1:** Demographic and clinical characteristics of patients, *n* = 135.

Characteristic	Number	Percentage, %
Age at initial MBM diagnosis, median (range), years	63 (19–87)	
Sex		
Male	76	56.3
Female	59	43.7
Date of diagnosis		
2011–2016	71	52.6
2017–2021	64	47.4
PS		
0	60	44.4
1	59	43.7
2	6	4.4
3	8	5.9
4	2	1.5
Neurologic symptoms		
Yes	75	55.6
No	60	44.4
Number of MBM		
Median (range)	2 (1–113)	
1	46	34.1
2–3	38	28.2
>=4	51	37.8
MBM size, mm^a^		
Median (range), mm	13 (1–74)	
<10	37	27.4
10–20	61	45.2
20–30	23	17.0
>30	14	10.4
Primary location		
Non‐acral cutaneous	72	53.3
Acral	29	21.5
Mucosal	22	16.3
Uveal	1	0.7
Unknown	11	8.2
Number of extracranial metastasis organs		
Median (range)	3 (0–8)	
<=3	75	55.6
> = 4	60	44.4
LDH, U/L		
Median (range)	246 (131–2840)	
<ULN	65	48.1
> = ULN	70	51.9
PD‐L1		
Unknown	114	84.4
>=1%	12	8.9
<1%	9	6.7
BRAF mutation		
Unknown	31	23.0
Yes (V600E)	29	20.7
Yes (V600K)	2	1.5
Yes (V600R)	1	0.7
Yes (Subtype unspecified)	18	14.1
No	54	40.0
Initial Systemic therapy		
None	21	15.6
Single‐agent ICI	64	47.4
COMBI‐ICI	17	12.6
TT	24	17.8
Chemotherapy	9	6.7
Radiotherapy		
None	0	0
STI	110	81.5
WBRT	25	18.5
Prior systemic therapy		
Single‐agent ICI	18	12.9
COMBI‐ICI	5	3.6
TT	10	7.1
Chemotherapy	2	1.4
None	109	77.9

Abbreviations: COMBI‐ICI, combination therapy of nivolumab and ipilimumab; ICI, immune checkpoint inhibitor; LDH, lactate dehydrogenase;MBM, melanoma brain metastasis; PS, performance status; STI, stereotactic irradiation; TT, targeted therapy; ULN, upper limit normal; WBRT, whole brain radiation therapys.

^a^ MBM size means the largest brain metastasis in each patient.

### 3.2 Survival of all patients.

**FIGURE 1:**
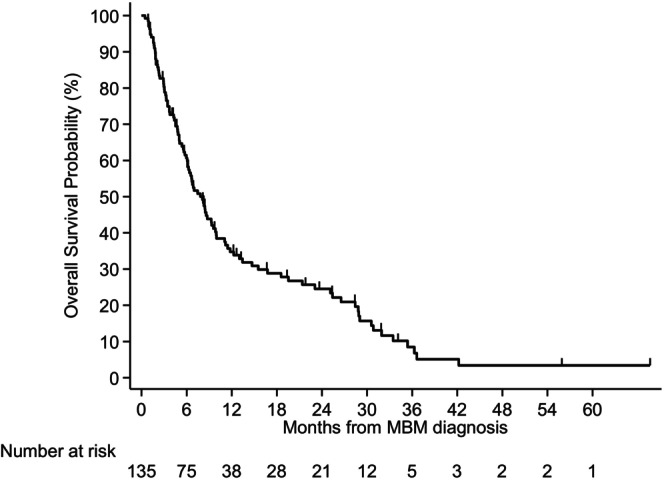
Kaplan–Meier analysis of overall survival (OS) of patients with Melanoma Brain Metastasis. MBM, melanoma brain metastasis.

Figure 1 shows the survival curve of all the patients included in our study. The median OS after an MBM diagnosis was 7.8 months (95% confidence interval [CI] 6.1–9.6). The 6‐month and 1‐year survival rates were 60.7% and 34.8%, respectively.

### 3.3 Prognostic factors

Table 2 presents the results of the Cox regression analysis. Ethnicity was excluded as a variable because of an imbalanced number of subgroups. On univariate analysis, presence of neurologic symptoms, fewer extracranial metastatic organs, low serum LDH levels, systemic therapy of single‐agent ICIs or TTs, and radiotherapy of STI significantly improved the outcomes. On multivariate analysis, non‐acral primary location, low serum LDH level, systemic therapy of single‐agent ICIs or TTs, and radiotherapy of STI significantly improved outcomes. Patients with acral melanoma as the primary location had shorter survival (hazard ratio [HR] 2.46, 95% confidence interval [CI] 1.21–4.98, *p* = 0.01). Patients with serum LDH levels higher than the upper limit of normal (ULN) had shorter survival than those with serum LDH levels lower than ULN (HR 3.84, 95% CI 2.01–7.36, *p* < 0.001). Patients who had received single‐agent ICI or TT had longer survival than those who had received no systemic therapy (single‐agent ICI: HR 0.27, 95% CI 0.12–0.61, *p* = 0.002; TT: HR 0.15, 95% CI 0.05–0.42, *p* < 0.001). Patients who received WBRT had shorter survival than those who received STI (HR 2.66, 95% CI 1.33–5.36, *p* = 0.006).

**TABLE 2:** Prognostic factors for Overall Survival: Cox regression analysis.

	Univariate Analysis	95% CI	*p* Value	Multivariate Analysis	95% CI	*p* Value
	HR			HR		
Age at initial MBM diagnosis, years						
<60	1 (Reference)			1 (Reference)		
>=60	0.78	0.53–1.15	0.21	0.89	0.50–1.56	0.67
Sex						
Male	1 (Reference)			1 (Reference)		
Female	0.97	0.66–1.43	0.89	1.06	0.61–1.83	0.84
Date of diagnosis						
2011–2016	1 (Reference)			1 (Reference)		
2017–2021	0.99	0.66–1.48	0.96	0.80	0.43–1.51	0.49
PS						
0	1 (Reference)			1 (Reference)		
>=1	0.80	0.55–1.18	0.26	1.71	0.90–3.25	0.10
Neurologic symptoms						
Yes	1 (Reference)			1 (Reference)		
No	1.62	1.09–2.40	**0.02**	1.63	0.97–2.73	0.07
Number of MBM						
1	1 (Reference)			1 (Reference)		
2–3	0.87	0.54–1.40	0.56	0.86	0.44–1.69	0.67
>=4	0.74	0.47–1.16	0.19	1.13	0.58–2.21	0.71
MBM size, mm^a^						
<10	1 (Reference)			1 (Reference)		
10–20	1.39	0.88–2.21	0.16	1.28	0.71–2.32	0.41
20–30	1.29	0.73–2.27	0.39	0.63	0.26–1.48	0.29
>30	0.63	0.30–1.33	0.22	0.75	0.29–1.95	0.56
Primary location						
Non‐acral cutaneous	1 (Reference)			1 (Reference)		
Acral	1.23	0.77–1.99	0.39	2.46	1.21–4.98	0.01
Mucosal	0.93	0.54–1.59	0.79	0.87	0.39–1.94	0.73
Unknown	1.67	0.84–3.30	0.14	2.35	0.88–6.31	0.09
Number of extracranial metastasis organs						
<=3	1 (Reference)			1 (Reference)		
>=4	1.73	1.17–2.54	**0.006**	1.59	0.92–2.76	0.10
LDH, U/L						
<ULN	1 (Reference)			1 (Reference)		
>=ULN	2.86	1.92–4.26	**<0.001**	3.84	2.01–7.36	<0.001
BRAF mutation						
Yes	1 (Reference)			1 (Reference)		
No	1.10	0.70–1.72	0.68	1.10	0.56–2.18	0.78
Initial systemic therapy						
None	1 (Reference)			1 (Reference)		
Single‐agent ICI	0.51	0.29–0.90	**0.02**	0.27	0.12–0.61	0.002
COMBI‐ICI	0.52	0.24–1.15	0.11	0.39	0.14–1.12	0.08
TT	0.34	0.17–0.69	**0.003**	0.15	0.05–0.42	<0.001
Chemotherapy	0.78	0.34–1.78	0.57	0.39	0.13–1.22	0.11
Radiotherapy						
STI	1 (Reference)			1 (Reference)		
WBRT	2.55	1.59–4.08	**<0.001**	2.66	1.33–5.36	0.006

Abbreviations: CI, confidence interval; COMBI‐ICI, combination therapy of nivolumab and ipilimumab; HR, hazard ratio; ICI, immune checkpoint inhibitor; LDH, lactate dehydrogenase; MBM, melanoma brain metastasis; PS, performance status; STI, stereotactic irradiation; TT, targeted therapy; ULN, upper limit normal; WBRT, whole‐brain radiation therapy.

Bold values mean statistically significant.

^a^ MBM size means the largest brain metastasis in each patient.

### 3.4 Effectiveness of systemic therapy

**FIGURE 2:**
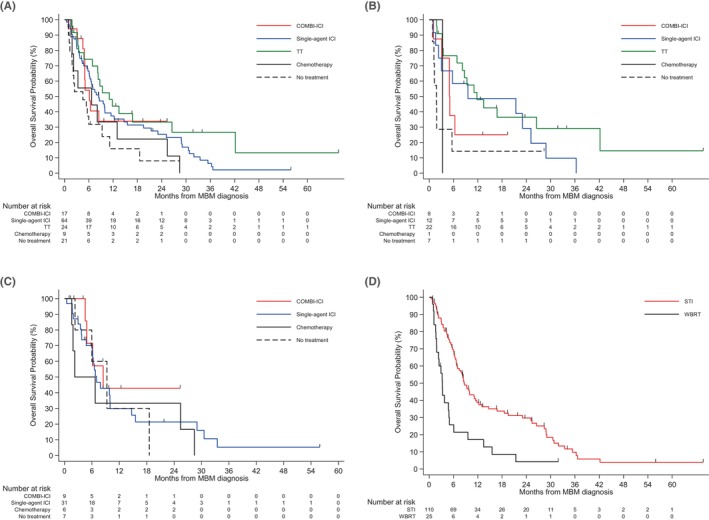
Survival curves categorized by regimens of initial systemic therapy and type of radiotherapy. (A) All patients: systemic therapy (B) BRAF mutant patients: systemic therapy (C) BRAF wild‐type patients: systemic therapy (D) all patients: radiotherapy. COMBI‐ICI, combination therapy of nivolumab and ipilimumab; ICI, immune checkpoint inhibitor; MBM, melanoma brain metastasis; STI, stereotactic irradiation; TT, targeted therapy; WBRT, whole‐brain radiation therapy.

Figure 2 shows the Kaplan–Meier curves comparing initial systemic therapy and radiotherapy. The median OS of patients treated with single‐agent ICI was 8.4 months (95% CI 6.3–11.5), and the 6‐month and 1‐year survival rates were 66.5% and 37.3%, respectively. The median OS of patients treated with COMBI‐ICI was 6.1 months (95% CI 4.9–not reached), and the 6‐month and 1‐year survival rates were 54.1% and 33.8%, respectively. The median OS of patients treated with TT was 11.1 months (95% CI 6.9–26.5), and the 6‐month and 1‐year survival rates were 74.2% and 43.7%, respectively. The median OS of patients treated with conventional chemotherapy was 6.7 months (95% CI 1.6–25.4), and the 6‐month and 1‐year survival rates were 55.6% and 33.3%, respectively. The median OS of patients not treated with systemic therapy was 4.5 months (95% CI 1.8–9.3), and the 6‐month and 1‐year survival rates were 31.8% and 15.9%, respectively. For all patients, there was no significant difference in survival curves between single‐agent ICI, the combination of nivolumab and ipilimumab, and TT (*p* = 0.33). In patients with BRAF‐mutant melanoma, there was no significant difference among single‐agent ICI, COMBI‐ICI, and TT (*p* = 0.23). For patients with BRAF wild‐type melanoma, there was no significant difference between single‐agent ICI and COMBI‐ICIs (*p* = 0.46). Concerning radiotherapy, the median OS of patients treated with STI was 8.6 months (95% CI 6.9–11.5) and the 6‐month and 1‐year survival rates were 68.6% and 38.7%, respectively. The median OS of patients treated with WBRT was 3.3 months (95% CI 1.9–4.9), and the 6‐month and 1‐year survival rate was 25.7% and 17.1%, respectively. There was a significant difference in survival curves between the STI and WBRT groups (*p* < 0.001).

Table 3 presents the characteristics of patients treated with ICI and TT, and Table 4 displays the results of the comparison of the effectiveness of each systemic therapy for MBM using Cox regression analysis. After adjusting for confounding factors, no difference in effectiveness was found between single‐agent ICI, COMBI‐ICI, and TT (COMBI‐ICI vs. single‐agent ICI, HR 0.71, 95% CI 0.27–1.88, *p* = 0.49; COMBI‐ICI vs. TT: HR 0.46, 95% CI 0.14–1.55, *p* = 0.21).

**TABLE 3:** Demographic and clinical characteristics of patients treated with immune checkpoint inhibitors and targeted therapy.

	COMBI‐ICI (*n* = 17)		Single‐agent ICI (*n* = 68)		TT (*n* = 24)		*p* Value
	Number	Percentage	Number	Percentage	Number	Percentage	
Age at initial MBM diagnosis, years							
<60	6	35.3	30	46.9	14	58.3	
> = 60	11	64.7	34	53.1	10	41.7	0.34
Sex							
Male	8	47.1	33	51.6	17	70.8	
Female	9	52.9	31	48.4	7	29.2	0.21
Date of diagnosis							
2011–2016	0	0	38	59.4	10	41.7	
2017–2021	17	100	26	40.6	14	58.3	**<0.001**
PS							
0	10	58.8	27	42.2	9	37.5	
> = 1	7	41.2	37	57.8	15	62.5	0.37
Neurologic symptoms							
Yes	8	47.1	37	57.8	12	50.0	
No	9	52.9	27	42.2	12	50.0	0.65
Number of MBM							
1	8	47.1	21	32.8	6	25.0	
>=2	3	17.7	18	28.1	6	75.0	
MBM size, mm^a^	6	35.3	25	39.1	12	50.0	0.61
<=10							
>10	8	58.8	17	26.6	7	29.2	
Primary location	7	41.2	27	42.2	11	45.8	
Non‐acral cutaneous	12	70.6	32	50	14	58.3	
Acral	2	11.8	10	15.6	9	37.5	
Mucosal	1	5.9	14	21.9	1	4.2	
Others	2	11.8	8	12.5	0	0	**0.03**
Number of extracranial metastatic organs							
<=3	8	47.1	34	53.1	16	66.7	
>3	9	52.9	30	46.9	8	33.3	0.40
LDH, U/L							
<ULN	8	47.1	29	45.3	17	70.8	
>=ULN	9	52.9	35	54.7	7	29.2	0.10
BRAF							
Unknown	0	0	21	32.8	1	4.2	
Yes	8	47.1	12	18.8	22	91.6	
No	9	52.9	31	48.4	1	4.2	**<0.001**
Radiotherapy							
STI	14	82.4	52	81.3	19	79.2	
WBRT	3	17.6	12	18.8	5	20.8	0.96
Prior systemic therapy							
COMBI‐ICI	0	0	1	1.6	2	8.3	
Single‐agent ICI	1	5.9	4	6.3	2	8.3	
TT	3	17.6	1	1.6	2	8.3	
Chemotherapy	0	0	0	0	0	0	
None	14	82.4	58	90.6	20	83.3	0.19

Abbreviations: COMBI‐ICI, combination therapy of nivolumab and ipilimumab; ICI, immune checkpoint inhibitor; LDH, lactate dehydrogenase; MBM melanoma brain metastasis; STI, stereotactic irradiation; TT, targeted therapy; ULN, upper limit normal; WBRT, whole brain radiation therapy.

Bold values mean statistically significant.

^a^ MBM size means the largest brain metastasis in each patient.

**TABLE 4:** Comparison of the effectiveness of systemic therapy in affecting Overall Survival: Cox regression analysis.

	Univariate Analysis	95% CI	*p* Value	Multivariate Analysis	95% CI	*p* Value
	HR			HR		
First‐line systemic therapy						
COMBI‐ICI	1 (Reference)					
Single‐agent ICI	0.99	0.50–1.97	0.99	0.71	0.27–1.88	0.49
TT	0.66	0.30–1.47	0.31	0.46	0.14–1.55	0.21
Age at initial MBM diagnosis, years						
<60	1 (Reference)					
>=60	0.69	0.45–1.08	0.1	0.8	0.40–1.60	0.53
Sex						
Male	1 (Reference)					
Female	1.06	0.68–1.64	0.8	1.08	0.57–1.94	0.88
Date of diagnosis						
2011–2016	1 (Reference)					
2017–2021	1.25	0.78–1.98	0.35	0.88	0.43–1.78	0.72
PS						
0	1 (Reference)					
>=1	0.8	0.51–1.25	0.33	2.01	0.90–4.49	0.09
Neurologic symptoms						
Yes	1 (Reference)					
No	1.54	0.98–2.42	0.06	1.7	0.90–3.18	0.1
Number of MBM						
1	1 (Reference)					
>=2	0.72	0.41–1.27	0.25	0.48	0.20–1.16	0.1
MBM size, mm^a^	0.71	0.42–1.19	0.19	0.76	0.34–1.69	0.5
<=10						
>10	1 (Reference)					
Primary location	1.42	0.85–2.38	0.18	1.76	0.89–3.49	0.11
Non‐acral cutaneous	1.5	0.79–2.86	0.22	1.08	0.42–2.79	0.87
Acral	0.57	0.23–1.38	0.21	0.8	0.26–2.46	0.69
Mucosal						
Others	1 (Reference)					
Number of extracranial metastatic organs	1.19	0.69–2.05	0.54	2.42	1.10–5.33	**0.03**
<=3	0.89	0.48–1.67	0.72	0.8	0.30–2.11	0.65
>3	1.89	0.87–4.07	0.11	1.84	0.65–5.21	0.25
LDH, U/L						
<ULN	1 (Reference)			1 (Reference)		
>=ULN	1.89	1.22–2.94	**0.005**	1.49	0.78–2.86	0.23
BRAF						
Yes	1 (Reference)			1 (Reference)		
No	2.92	1.86–4.59	**<0.001**	3.75	1.81–7.79	**<0.001**
Radiotherapy						
STI	1 (Reference)					
WBRT	1.2	0.72–1.99	0.48	1.41	0.61–3.25	0.42

Abbreviations: CI, confidence interval; COMBI‐ICI, combination therapy of nivolumab and ipilimumab; HR, hazard ratio; ICI, immune checkpoint inhibitor; LDH, lactate dehydrogenase; ULN, upper limit normal; STI, stereotactic irradiation; TT, targeted therapy; MBM, melanoma brain metastasis; WBRT, whole brain radiation therapy.

Bold values mean statistically significant.

^a^ MBM size means the largest brain metastasis in each patient.

## 4 DISCUSSION

In this study, we retrospectively investigated the survival of patients with MBM and associated factors. Consequently, Asian patients with MBM have a shorter prognosis than Caucasian patients, as discussed in the following section. To the best of our knowledge, this is the first study to present the possibility that COMBI‐ICI is not superior to ICI and TT when treating Asian patients with MBM.

According to previous studies, owing to recent improvements in chemotherapy and radiotherapy, the OS of patients with MBM has increased from approximately 4–14 months.^2^, ^10^ However, our study revealed that the outcomes of patients with MBMs in Japan did not improve as significantly as reported in other studies. Previous studies of Asian patients with MBMs showed a median OS of 7–8 months, which is comparable to our findings.^11^, ^12^ Although previous studies have shown limited effectiveness of ICI and TT in Asian patients with unresectable melanoma, Asian patients with MBMs may also respond poorly to these systemic therapies.^8^

The associated factors identified in multivariate analysis were primary location, serum LDH levels, type of initial systemic therapy, and type of radiotherapy. Univariate analysis identified presence of neurologic symptoms as being associated with higher overall survival, but this was not observed during multivariate analysis, indicating that this result could be a statistical artifact possibly due to the small sample size or confounding factors.

It has been reported that ICIs are less effective against acral and mucosal melanomas, possibly because their somatic mutation burden is lower than that in non‐acral cutaneous melanomas.^13^, ^14^, ^15^, ^16^ The results of our study suggest that the prognosis of MBM is also shorter when the subtype is acral melanoma, although mucosal melanoma has no shorter survival time than non‐acral melanoma. Serum LDH levels have been reported as a prognostic factor.^17^, ^18^, ^19^ The American Joint Committee on Cancer (AJCC) 8th edition articulates that LDH levels affect the TNM classification.^20^

We found no statistical difference in survival between patients with BRAF wild‐type and BRAF mutant melanoma. It has been reported that patients with a V600K mutation may respond better to ICIs compared to those with a V600E mutation. However, in our study, all patients with BRAF mutant melanoma were treated with TTs as first‐line therapy, so our results cannot support this hypothesis.^21^

Our study demonstrated that patients treated with ICI or TT had longer survival than those treated with conventional chemotherapy or those not treated with systemic therapy. In addition, there was no difference between the effectiveness of single‐agent ICI, COMBI‐ICIs, and TT, regardless of *BRAF* ^V600^ mutational status. The effectiveness of ICI and TT for unresectable melanoma has been demonstrated in many studies involving Caucasian cohorts, with limited effectiveness demonstrated in studies involving Asian cohorts.^9^, ^22^, ^23^ While TT is only effective for BRAF‐mutated melanoma, previous studies have demonstrated that COMBI‐ICI is more effective for unresectable melanoma than TT and single‐agent ICI, regardless of *BRAF* ^V600^ mutational status.^24^, ^25^ Even when we focus on patients with MBM, those treated with COMBI‐ICIs have longer survival times than those treated with single‐agent ICI and TT.^4^, ^5^, ^6^, ^7^, ^8^ Based on these studies, it was recently recommended that COMBI‐ICI be used for patients with MBM, regardless of *BRAF* ^V600^ mutational status. However, the evidence underlying this recommendation is that studies on Caucasian cohorts and Asian patients are rare. Our study suggests that COMBI‐ICIs might not be superior to ICI and TT when treating Asian patients with MBM, and that there may be a need to re‐assess the recommendation of COMBI‐ICIs as the first‐line therapy in treating Asian patients with MBM.

Next, we discuss the local treatment. Our study demonstrated that patients treated with STI had longer survival than those treated with WBRT. Previous studies have demonstrated the effectiveness of STI over WBRT.^11^ In general, STIs are preferred for up to four brain metastases.^26^ On the other hand, WBRT is preferred when there are more than five metastases, or when the size is over 3 cm. However, there was no difference in the number and size of MBM between patients who underwent STI and those who underwent WBRT in this study. Our results suggest the potential of STI in treating multiple metastases.

A major limitation of our study was the inherent potential bias of retrospective studies. Many of our patients had received systemic therapy for melanoma before being diagnosed with MBM, and they also received concurrent radiotherapy while being treated with first‐line therapies. These other treatments could have affected the effectiveness of the first‐line therapies. In addition, the number of patients with MBM treated with COMBI‐ICI was relatively small, making the comparison between each treatment group less reliable. Furthermore, the follow‐up period was short making it difficult to assess response durability. Therefore, further multicenter analyses and prospective studies with larger cohorts are required to confirm the efficacy of systemic therapy in Asian patients with MBM.

## 5 CONCLUSIONS

We retrospectively investigated the survival of patients with MBMs at our facility. The survival of Asian patients with MBM is poor. ICI, TT, and STI were associated with improved survival; however, we found no significant superiority of COMBI‐ICI over single‐agent ICI or TT in terms of effectiveness. Further investigations using larger cohorts, multicenter analyses, and prospective studies are necessary to assess the effectiveness of systemic therapy in Asian patients with MBM.

## AUTHOR CONTRIBUTIONS

**Shogo Wada:** Formal analysis (lead); methodology (lead); software (lead); validation (lead); visualization (lead); writing – original draft (lead). **Dai Ogata:** Conceptualization (lead); data curation (lead); formal analysis (supporting); investigation (lead); methodology (supporting); project administration (lead); resources (lead); writing – review and editing (lead). **Tairo Kashihara:** Resources (supporting); writing – review and editing (supporting). **Kae Okuma:** Resources (supporting); writing – review and editing (supporting). **Hirofumi Eto:** Data curation (supporting); investigation (supporting); resources (supporting); visualization (supporting); writing – review and editing (supporting). **Eiji Nakano:** Resources (supporting); writing – review and editing (supporting). **Akira Takahashi:** Resources (supporting); writing – review and editing (supporting). **Kenjiro Namikawa:** Resources (supporting); writing – review and editing (supporting). **Hiroshi Igaki:** Funding acquisition (supporting); resources (supporting); supervision (supporting); writing – review and editing (supporting). **Naoya Yamazaki:** Conceptualization (supporting); funding acquisition (lead); project administration (supporting); resources (supporting); supervision (lead); writing – review and editing (supporting).

## FUNDING INFORMATION

This work was partially supported by the National Cancer Center Research and Development Fund (grant number 2023‐J‐3).

## CONFLICT OF INTEREST STATEMENT

The authors declare no conflict of interest.

## Data Availability

Data available on request due to privacy/ethical restrictions.

## References

[1] Schouten LJ , Rutten J , Huveneers HA , Twijnstra A . Incidence of brain metastases in a cohort of patients with carcinoma of the breast, colon, kidney, and lung and melanoma. Cancer. 2002;94:2698‐2705. 12173339 10.1002/cncr.10541

[2] Fife KM , Colman MH , Stevens GN , et al. Determinants of outcome in melanoma patients with cerebral metastases. J Clin Oncol. 2004;22:1293‐1300. 15051777 10.1200/JCO.2004.08.140

[3] Wolf A , Zia S , Verma R , et al. Impact on overall survival of the combination of BRAF inhibitors and stereotactic radiosurgery in patients with melanoma brain metastases. J Neurooncol. 2016;127:607‐615. 26852222 10.1007/s11060-016-2072-6

[4] Tawbi HA , Forsyth PA , Algazi AP , et al. Combined nivolumab and ipilimumab in melanoma metastatic to the brain. N Engl J Med. 2018;379:722‐730. 30134131 10.1056/NEJMoa1805453 PMC8011001

[5] Tawbi HA , Forsyth PA , Hodi FS , et al. Long‐term outcomes of patients with active melanoma brain metastases treated with combination nivolumab plus ipilimumab (CheckMate 204): final results of an open‐label, multicentre, phase 2 study. Lancet Oncol. 2021;22:1692‐1704. 34774225 10.1016/S1470-2045(21)00545-3 PMC9328029

[6] Hilbers ML , Dimitriou F , Lau P , et al. Real‐life data for first‐line combination immune‐checkpoint inhibition and targeted therapy in patients with melanoma brain metastases. Eur J Cancer. 2021;156:149‐163. 34454317 10.1016/j.ejca.2021.07.028

[7] Long GV , Atkinson V , Lo S , et al. Combination nivolumab and ipilimumab or nivolumab alone in melanoma brain metastases: a multicentre randomised phase 2 study. Lancet Oncol. 2018;19:672‐681. 29602646 10.1016/S1470-2045(18)30139-6

[8] Yamazaki N , Takenouchi T , Nakamura Y , et al. Prospective observational study of the efficacy of nivolumab in Japanese patients with advanced melanoma (creative study). Jpn J Clin Oncol. 2021;51:1232‐1241. 34115870 10.1093/jjco/hyab064 PMC8326387

[9] Bai X , Shoushtari AN , Betof Warner A , et al. Benefit and toxicity of programmed death‐1 blockade vary by ethnicity in patients with advanced melanoma: an international multicentre observational study. Br J Dermatol. 2022;187:401‐410. 35293617 10.1111/bjd.21241

[10] Hasanov M , Milton DR , Bea Davies A , et al. Changes in outcomes and factors associated with survival in melanoma patients with brain metastases. Neuro Oncol. 2022;25:noac251. 10.1093/neuonc/noac251 PMC10326492 36510640

[11] Matsunaga S , Shuto T , Yamamoto M , et al. Gamma knife radiosurgery for metastatic brain tumors from malignant melanomas: a Japanese multi‐institutional cooperative and retrospective cohort study (JLGK1501). Stereotact Funct Neurosurg. 2018;96:162‐171. 29969770 10.1159/000489948

[12] Wang Y , Lian B , Si L , et al. Real‐world analysis of clinicopathological characteristics, survival rates, and prognostic factors in patients with melanoma brain metastases in China. J Cancer Res Clin Oncol. 2021;147:2731‐2740. 33611636 10.1007/s00432-021-03563-0 PMC11802154

[13] Curtin JA , Busam K , Pinkel D , Bastian BC . Somatic activation of KIT in distinct subtypes of melanoma. J Clin Oncol. 2006;24:4340‐4346. 16908931 10.1200/JCO.2006.06.2984

[14] Nakamura Y , Namikawa K , Yoshino K , et al. Anti‐PD1 checkpoint inhibitor therapy in acral melanoma: a multicenter study of 193 Japanese patients. Ann Oncol. 2020;31:1198‐1206. 32522691 10.1016/j.annonc.2020.05.031

[15] Nakamura Y , Namikawa K , Yoshikawa S , et al. Anti‐PD‐1 antibody monotherapy versus anti‐PD‐1 plus anti‐CTLA‐4 combination therapy as first‐line immunotherapy in unresectable or metastatic mucosal melanoma: a retrospective, multicenter study of 329 Japanese cases (JMAC study). ESMO Open. 2021;6:100325. 34839104 10.1016/j.esmoop.2021.100325 PMC8633880

[16] Nakamura Y , Namikawa K , Kiniwa Y , et al. Efficacy comparison between anti‐PD‐1 antibody monotherapy and anti‐PD‐1 plus anti‐CTLA‐4 combination therapy as first‐line immunotherapy for advanced acral melanoma: a retrospective, multicenter study of 254 Japanese patients. Eur J Cancer. 2022;176:78‐87. 36194906 10.1016/j.ejca.2022.08.030

[17] Long GV , Trefzer U , Davies MA , et al. Dabrafenib in patients with Val600Glu or Val600Lys BRAF‐mutant melanoma metastatic to the brain (BREAK‐MB): a multicentre, open‐label, phase 2 trial. Lancet Oncol. 2012;13:1087‐1095. 23051966 10.1016/S1470-2045(12)70431-X

[18] McArthur GA , Maio M , Arance A , et al. Vemurafenib in metastatic melanoma patients with brain metastases: an open‐label, single‐arm, phase 2, multicentre study. Ann Oncol. 2017;28:634‐641. 27993793 10.1093/annonc/mdw641

[19] Bander ED , Yuan M , Carnevale JA , et al. Melanoma brain metastasis presentation, treatment, and outcomes in the age of targeted and immunotherapies. Cancer. 2021;127:2062‐2073. 33651913 10.1002/cncr.33459 PMC9275782

[20] Gershenwald JE , Scolyer RA , Hess KR , et al. Melanoma staging: evidence‐based changes in the American joint committee on cancer eighth edition cancer staging manual. CA Cancer J Clin. 2017;67:472‐492. 29028110 10.3322/caac.21409 PMC5978683

[21] Nepote A , Avallone G , Ribero S , et al. Current controversies and challenges on BRAF V600K‐mutant cutaneous melanoma. J Clin Med. 2022;11:828. 35160279 10.3390/jcm11030828 PMC8836712

[22] Inozume T , Namikawa K , Kato H , et al. Analyzing the relationship between the efficacy of first‐line immune checkpoint inhibitors and cumulative sun damage in Japanese patients with advanced BRAF wild‐type nonacral cutaneous melanoma: a retrospective real‐world, multicenter study. J Dermatol Sci. 2023;110:19‐26. 37045720 10.1016/j.jdermsci.2023.03.008

[23] Ahmed KA , Abuodeh YA , Echevarria MI , et al. Clinical outcomes of melanoma brain metastases treated with stereotactic radiosurgery and anti‐PD‐1 therapy, anti‐CTLA‐4 therapy, BRAF/MEK inhibitors, BRAF inhibitor, or conventional chemotherapy. Ann Oncol. 2016;27:2288‐2294. 27637745 10.1093/annonc/mdw417 PMC8890451

[24] Wolchok JD , Chiarion‐Sileni V , Gonzalez R , et al. Long‐term outcomes with nivolumab plus ipilimumab or nivolumab alone versus ipilimumab in patients with advanced melanoma. J Clin Oncol. 2022;40:127‐137. 34818112 10.1200/JCO.21.02229 PMC8718224

[25] Atkins MB , Lee SJ , Chmielowski B , et al. Combination dabrafenib and trametinib versus combination nivolumab and ipilimumab for patients with advanced BRAF‐mutant melanoma: the DREAMseq trial‐ECOG‐ACRIN EA6134. J Clin Oncol. 2023;41:186‐197. 36166727 10.1200/JCO.22.01763 PMC9839305

[26] Rauschenberg R , Bruns J , Brütting J , et al. Impact of radiation, systemic therapy and treatment sequencing on survival of patients with melanoma brain metastases. Eur J Cancer. 2019;110:11‐20. 30739835 10.1016/j.ejca.2018.12.023

